# Application of the PAMONO-Sensor for Quantification of Microvesicles and Determination of Nano-Particle Size Distribution

**DOI:** 10.3390/s17020244

**Published:** 2017-01-27

**Authors:** Victoria Shpacovitch, Irina Sidorenko, Jan Eric Lenssen, Vladimir Temchura, Frank Weichert, Heinrich Müller, Klaus Überla, Alexander Zybin, Alexander Schramm, Roland Hergenröder

**Affiliations:** 1 Leibniz Institute für Analytische Wissenschaften, ISAS e.V., Bunsen-Kirchhoff-Straße 11, 44139 Dortmund, Germany; alexander.zybin@isas.de (A.Z.); roland.hergenroeder@isas.de (R.H.); 2 MIVITEC GmbH, Wamslerstraße.4, 81829 Munich, Germany; irina.sidorenko1104@googlemail.com; 3 Department of Computer Science VII, TU Dortmund University, Otto-Hahn-Straße. 16, 44227 Dortmund, Germany; janeric.lenssen@tu-dortmund.de (J.E.L.); frank.weichert@tu-dortmund.de (F.W.); heinrich.mueller@tu-dortmund.de (H.M.); 4 Institute of Clinical and Molecular Virology, University Hospital Erlangen, Friedrich-Alexander University Erlangen-Nürnberg, Schlossgarten 4, 91054 Erlangen, Germany; Vladimir.Temchura@viro.med.uni-erlangen.de (V.T.); klaus.ueberla@fau.de (K.Ü.); 5 Children’s Hospital, Oncology Laboratory, University Clinic Essen, Hufelandstraße. 55, 45122 Essen, Germany; alexander.schramm@uk-essen.de

**Keywords:** plasmonic sensors, surface plasmon resonance, extracellular vesicles, microvesicles, machine learning, deep learning

## Abstract

The PAMONO-sensor (plasmon assisted microscopy of nano-objects) demonstrated an ability to detect and quantify individual viruses and virus-like particles. However, another group of biological vesicles—microvesicles (100–1000 nm)—also attracts growing interest as biomarkers of different pathologies and needs development of novel techniques for characterization. This work shows the applicability of a PAMONO-sensor for selective detection of microvesicles in aquatic samples. The sensor permits comparison of relative concentrations of microvesicles between samples. We also study a possibility of repeated use of a sensor chip after elution of the microvesicle capturing layer. Moreover, we improve the detection features of the PAMONO-sensor. The detection process utilizes novel machine learning techniques on the sensor image data to estimate particle size distributions of nano-particles in polydisperse samples. Altogether, our findings expand analytical features and the application field of the PAMONO-sensor. They can also serve for a maturation of diagnostic tools based on the PAMONO-sensor platform.

## 1. Introduction

The PAMONO-sensor (plasmon assisted microscopy of nano-objects) harnesses a surface plasmon resonance (SPR) phenomena for the imaging of biological and non-biological nano-objects [1,2]. Surface plasmon resonance is a label-free optical analytical technique, which enables real-time studies of interactions between biomolecules immobilized onto sensor surface and their solubilized counterparts (protein-DNA, DNA-DNA, protein-protein, etc.) [3,4,5,6]. In the conventional SPR approach, the layers of biomolecules formed on a gold sensor surface alter the refractive conditions near the sensor surface. These alterations serve as a basis for the real-time measurements of the binding efficiency of biomolecules during determination of affinity constants or concentration measurements. For a long time, plasmonic sensors were not deemed as effective instruments for the quantification of the binding of individual nano-scale particles to the sensor surface. Only recently, results from independent research teams proved the applicability of SPR-based sensors in studies of biological nano-objects [1,2,7,8]. The PAMONO-sensor was demonstrated to be an appropriate tool for detection and quantification of individual viruses, virus-like particles and inorganic nano-particles [9]. It is also not surprising that SPR-based sensors were recently [10,11] tried out in studies focused on detection and quantification of another group of biological nano-vesicles—extracellular vesicles. Extracellular vesicles (EVs) are cell-derived vesicles, which serve as a means of protected intercellular communication and information exchange within an organism [12]. Non-apoptotic EVs can carry different biologically active cargo and may be divided according to their size into two groups: exosomes (20–100 nm) and microvesicles (MVs) (100–1000 nm) [12,13].

However, commonly used techniques for quantification and characterization of EVs—flow cytometry and nano-particle tracking analysis—have certain limitations. Both methods require labeling prior to specific detection of EVs. Moreover, flow cytometry suffers from resolution and sensitivity problems caused by the size of EVs [14]. The use of SPR-based sensor in the groundbreaking work of Im and collegues [10] proved in general the power of plasmonic sensors in the detection and quantification of EVs. However, an advanced and painstaking approach harnessed by the authors for the preparation of sensor surface (focused ion beam milling for the preparation of nano-holes) makes the described technique time-consuming and expensive. A conventional SPR method employed by Zhu and co-workers [11] deals with a formation of a layer of EVs onto the sensor surface. Thus, complex calculations are required for the determination of particle concentrations using the thickness of the formed layer. Moreover, a monitoring of integral intensity changes exclude an opportunity to analyze vesicle size distribution in the mixture of vesicles, which is known to be an important characteristic of EV samples. The PAMONO-sensor enables a real-time imaging of the binding of single biological label-free submicrometer-vesicles to the functionalized sensor surface and does not require advanced technology for the preparation of sensor slides. Thus, we reasoned that the PAMONO-sensor can overcome constraints mentioned above, and we were inspired to investigate the detection and quantification of microvesicles (MVs) using this sensor.

For this purpose, we utilize cysteine-conjugated protein A/G functionalization of our PAMONO-sensor, which permits achieving an oriented immobilization of unmodified anti-target antibody. We investigated whether protein A/G functionalization is applicable for detection and quantification of MVs using the PAMONO-sensor. We also studied whether a protein A/G functionalized sensor slide employed in a PAMONO-sensor would be re-usable after the elution of a target-capturing antibody layer. In the proof-of-principal experiments, we show a possibility to perform particle size distribution analysis using the PAMONO-sensor measurements. For this purpose, we applied novel machine learning methods for image processing. Here, we describe how we train a parametrized model that receives images of SPR waves and is able to estimate a particle size for the particle triggering the alteration of reflection. Moreover, we are able to derive particle size distributions for whole samples and compare them with distributions measured by a reference method.

## 2. Materials and Methods

In the Section 2.1, we depict a scheme of the surface plasmon resonance experimental setup employed in the PAMONO-sensor. The Section 2.2 provides information about chemicals used in our study as well as about the preparation procedures of biological nano-vesicles (microvesicles and virus-like particles) utilized in our work. Section 2.3 and Section 2.4 describe the measurement conditions for biological and polystyrene particles, respectively. The application of machine learning techniques for monitoring of particle size distribution is presented in Section 2.5.

### 2.1. Surface Plasmon Resonance (SPR) Experimental Setup

**Figure 1:**
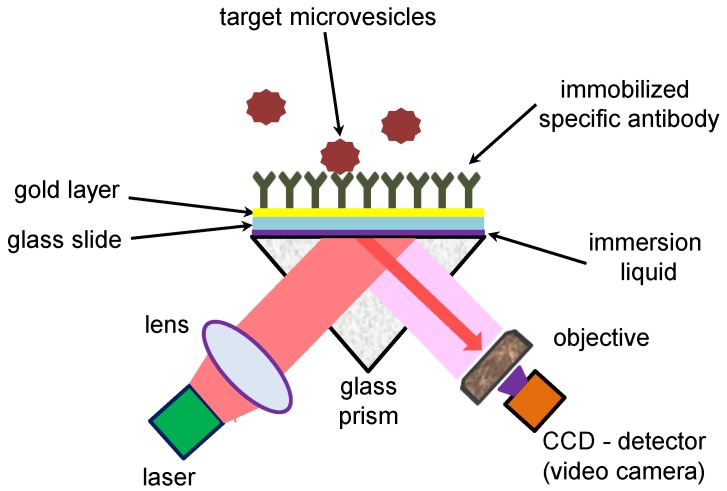
Scheme of PAMONO-sensor experimental setup used for detection of biological nano-vesicles.

The PAMONO-sensor permits the imaging of biological nano-vesicles utilizing a Kretschmann’s scheme [15] of plasmon excitation with an illumination of a gold sensor surface via a glass prism. The schematic view of the PAMONO-sensor set up is presented in Figure 1 and is also described in [1,2]. In contrast to conventional SPR, the PAMONO-sensor employs local changes of reflectivity in order to image the binding events of individual biological or inorganic nano-particles [16].

Glass slides were covered with titanium and gold layer (approximate gold thickness: 45 nm) (PHASIS, Geneva, Switzerland). A magnetron-sputtering technique was employed by PHASIS for the metal layer deposition. These glass slides are further referred to as “gold sensors”. During the experiments, a gold sensor is attached, by means of an immersion liquid, to a glass prism and illuminated through the glass prism by a collimated beam of a diode laser (HL6750MG, Thorlabs GmbH, Dachau, Germany) (Figure 1). The excitation wavelength of the incident beam is *λ* ≈ 675 nm. The incidence angle is chosen on the left (smaller angle) slope of the resonance minimum. A 50 mm Minolta Rokkor MD (Minolta, Osaka, Japan) photo-objective with an aperture of 1/1.7 is used to image the gold surface onto a 5-megapixel GC2450 Prosilica video camera (Allied Vision Technologies GmbH, Stadtroda, Germany) equipped with Sony ICX625 CCD (charge-coupled device)- image sensor (3.45 μm × 3.45 μm pixel size). A magnification factor of ≈6 is chosen, so that one pixel corresponds to approximately 0.6 μm on the sensor surface. The PAMONO-sensor utilizes an U-shaped flow-cell system in the current work. The flow-cell chamber has the following dimensions: 10.5 mm × 13 mm × 1.3 mm (width × length × depth). The width of a channel in a U-shaped flow system is ≈2 mm. Biological vesicles are pumped through the flow-cell as a suspension in Phosphate-buffered saline (PBS) at a flow rate of 0.3 mL/min. A LM10 device (Malvern Instruments Ltd., Malvern, UK via Malvern Instruments GmbH, Herrenberg, Germany) helps to verify the concentration of biological and inorganic vesicles and particles used in the presented study. The applied version of the LM10 device utilizes a blue laser with wavelength λ ≈ 405 nm and a camera produced by Hamamatsu Photonics (C11440-50B; A11893-02) (distributor in Germany: Hamamatsu Photonics Deutschland GmbH, Herrsching am Ammersee, Germany) for image recording. A used LM10 device is not equipped with fluorescent filters. This device performs size measurements and quantification of nano-particles based on light scattering and Brownian motion (nano-particle tracking analysis or NTA).

*Streampix 3.0* (NorPix, Montreal, Quebec, Canada) software is employed to record images. Furthermore, images are processed using the software created by the authors and described in details [17,18,19].

### 2.2. Chemical and Biological Materials: Preparation of Human Immunodeficiency Virus (HIV) Virus-Like Particles (VLPs) and Microvesicles (MVs) from SH SY5Y Neuroblastoma Cells

Phosphate-buffered saline (PBS) buffer was purchased from GE Healthcare (Pasching, Austria). Fetal Bovine Serum (FBS) Gold (Cat. A 15-151) for the experiments with SH-SY5Y cells was purchased from PAA Laboratories GmbH (now a part of GE Healthcare, Pasching, Austria). An exosome-free FBS used for the culture of SH-SY5Y cells was prepared from the purchased FBS Gold by ultracentrifugation. Human SH-SY5Y neuroblastoma cells were cultivated in RPMI1640 medium containing 10% FBS and antibiotics. Cells were authenticated by Short Tandem Repeats (STR) genotyping. Cell culture medium for HEK293T cells was based on Dulbecco’s modified Eagle Medium (Gibco by Life Technologies, Darmstadt, Germany) supplied with 10% FBS (Invitrogen, Karlsruhe, Germany) and antibiotics: penicillin and streptomycin (both from Life Technologies, Darmstadt, Germany). Sucrose was received from AppliChem GmbH (Darmstadt, Germany). Tubes and pipettes were purchased from Eppendorf (Wesseling-Berzdorf, Germany). Other reagents were purchased from Bio-Techne (Wiesbaden-Nordenstadt, Germany), RatioLab (Dreieich, Germany), Behr Labor-Technik (Düsseldorf, Germany), Sigma-Aldrich (Schnelldorf, Germany) and VWR International GmbH (Darmstadt, Germany).

HIV-VLPs were produced in HEK293T human cell-line as described in details previously [20,21]. To pseudotype VLPs with a model antigen, the cells were co-transfected with the plasmid encoding surface-anchored ovalbumin (OVA) [22]. To produce control VLPs, the cells were co-transfected with Carrier DNA (Invitrogen, Karlsruhe, Germany) resulting in VLPs carrying all producer-cell-derived surface proteins but lacked OVA. The VLPs were purified from the cell-culture medium and concentrated by ultracentrifugation through a 20% sucrose cushion [20]. Literature reports state that, HIV-VLPs are usually round-shaped nano-objects with size around 100–140 nm [23,24].

For microvesicle analysis, SH-SY5Y cells were cultivated in exosome-free FBS. SH-SY5Y parental cells or subclones thereof stably expressing either of the neurotrophin receptors, TrkA or TrkB were created as described in [25] and further used for purification of microvesicles (MVs). Supernatants were collected from sub-confluent cells. MVs were harvested from these supernatants by ultracentrifugation (100,000× *g*, 2 h 10 min, 4 °C). Presence of MVs was verified in these supernatants by Fluorescence-activated Cell Sorting (FACS) analysis using anti-CD9 or anti-CD81 antibody.

### 2.3. PAMONO Measurements of MVs Derived from SH-SY5Y Cells and Human Immunodeficiency Virus (HIV)-VLPs Produced by HEK293T Cells

**Figure 2:**
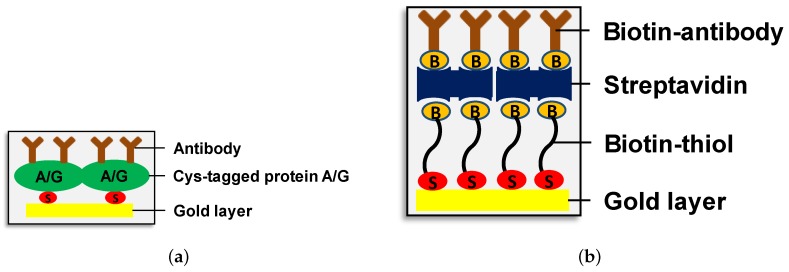
Schemes of the sensor surface functionalization approaches utilized in PAMONO-sensor. Cystein-conjugated protein A/G—antibody (without any conjugated tag) (**a**) and biotin-thiol—streptavidin—biotinylated antibody (**b**) self assembling monolayers can be formed on the gold sensor surface to capture the vesicles of interest.

To analyze MVs and HIV-VLPs, the gold sensor surface was functionalized under flow conditions. The ways of sensor functionalization are illustrated in Figure 2. A freshly prepared mixture of two parts of concentrated sulfuric acid and one part of 30% cold hydrogen peroxide solution was applied to clean the sensor surface before the functionalization step. The following self-assembling layers were formed: (1) the first layer is formed by cysteine-conjugated protein A/G (Bio-Techne, Wiesbaden-Nordenstadt, Germany) in a concentration of 30 μg/mL; (2) the second layer is constituted by anti-target antibody: anti-ovalbumin (anti-OVA) antibody (Biomol GmbH, Hamburg, Germany) in a concentration of 10 μg/mL for experiments with HIV-VLPs or anti-CD81 antibody (BD Pharmingen via Becton Dickinson GmbH, Heidelberg, Germany) in a concentration of 10 μg/mL to capture MVs derived from SH-SY5Y cells. The saturation stage of a monolayer formation is indicated by a uniform and temporal constant background image. If necessary, the elution of antibody layer from protein A/G layer is performed via application of IgG elution buffer (Thermo Fisher Scientific GmbH, Germany, www.thermofisher.com) under flow conditions. To evaluate selectivity, two types of HIV-VLPs—with and without OVA expressed on the particle surface—are used. Sensor functionalization with protein A/G and anti-OVA antibody is accomplished as described above (Figure 2a). In selectivity studies, the formation of other self-assembling layers was used in parallel onto another sensor slide (Figure 2b). This approach is applied in order to compare previously used biotin-thiol-based sensor functionalization with a novel protein A/G functionalization. The following layers are formed under flow conditions: (1) the first layer is biotin-thiol (HSC11-EG3 Biotin, ProChimia Surfaces Sp., 81-823 Sopot, Poland, www.prochimia.com) diluted with 11-mercapto-1-undecanol in a ratio 1:10; (2) the second layer consists of 2 μM streptavidin dissolved in PBS and functionalization was performed using 2 μM solution in PBS; and a (3) the third layer was formed by specific biotinylated rabbit anti-OVA antibody (Biomol GmbH, Hamburg, Germany) employed in a concentration of 10 μg/mL.

Image recording speed was 39 frames and 41 frames per second during the experiments with MVs from SH-SY5Y cells, and, in the experiments, investigating a possibility of the repeated use of a gold sensor, respectively. In the selectivity studies, the applied recording speed varied between 34–44 frames per second. However, during each experiment, image recording speed was kept constant.

### 2.4. PAMONO Measurements of Particle Size Distribution in the Mixtures of Particles

For these measurements, polystyrene nano-particles of different sizes are employed as a model system. Particles (100 nm, 200 nm, 300 nm) were purchased from Molecular Probes (a part of Life Technologies, Darmstadt, Germany). According to manufacturer’s instructions, the variation in particle diameter was ≈5% for 200 nm and 300 nm particles, and ≈8% for 100 nm particles. Gold sensor slides used in these measurements were covered with “Nüscoflock” in order to facilitate particle attachment to the sensor surface. “Nüscoflock” is a liquid containing 10% aluminum hydroxide chloride and purchased from Dr. Nüsgen Chemie (Kamen, Germany). The liquid was filtrated before application on the gold sensor surface. In order to obviate the formation of particle aggregates, nano-particles were incubated in an ultrasonic water-bath Elmasonic S10H (Elma, Singen, Germany) before PAMONO and LM10 measurements. Particles were pumped through the U-shaped flow-cell as a suspension in distilled water containing 0.075% sodium chloride. Image recording speed varied between 41–43 frames per second. However, it was kept constant during each experiment.

### 2.5. The Demonstration of Suitability of Convolutional Neural Networks for Real-Time Estimation of Nano-Particle Size Distributions

In order to expand the analytical abilities of the PAMONO-sensor, we investigated a possibility to apply Convolutional Neural Networks for the real-time estimation of nano-particle size distributions. In the current proof-of-principle studies, we used model particles (polystyrene nano-particles) for the training of neural networks. This approach is certainly useful at the initial level of neural network training, but it has to be further adopted for the data received from the mixtures of microvesicles. An appropriate “calibration” of the tested neural networks with biological nano-vesicles (virus-like particles of different sizes, for example) will be certainly required in experiments with microvesicles. The PAMONO-sensor provides image sequences containing a plasmon wave signal for each bound particle, which can be analyzed using methods from image processing. Given the captured images from the samples described in Section 2.4, we estimate particle size distributions for a whole sensor image sequence by using the following pipeline: first, the constant background of the images is eliminated, followed by the detection and extraction of plasmon wave signals from the image [19]. Second, the extracted signals are classified using a previously trained convolutional neural network model, which is detailed below. The neural networks output leads to size estimation for the input nano-particle. To produce the output distribution, we count the particles of different sizes and create a histogram with a bin size of 10 nm for the whole sequence. The whole pipeline is real-time capable, processing up to 45 frames per second on current generation graphics processing units.

As outlined by [1], the intensity of plasmon wave signals depends on the refractive index of the particle as well as the particle size. Therefore, a particle size can be inferred from intensity if the refractive index of the measured particles is known and constant. However, we found that the accurate, automatic extraction of an intensity value by applying methods from image processing provides difficulties due to distortions and noise in the sensor data. We achieved better results using a convolutional neural network model for nano-particle size classification, which learns the intensity extraction procedure. The limitation is that a model that was trained using particles of a specific refractive index can not be applied to classify the size of particles with different index values. Therefore, for each small interval of refractive indexes, an individual model has to be provided, which is achieved by training the model with the results of four calibration measurements (using particle suspensions with known sizes).

Artificial neural networks are universal function approximators (c.f. [26]) that receive an input vector $x\in R^{m}$ and compute a function $f\left(x\right)\in R^{n}$. During training of an artificial neural network, a large number of function parameters is iteratively changed to minimize a loss function L(f(x),y)∈R given ground truth vectors $y\in R^{n}$ for each input example vector x [27]. In the last few years, a special kind of network, convolutional neural networks (CNNs), achieved award-winning performance in several image and signal processing tasks [28,29]. They are feed-forward artificial neural networks for image inputs x, enhanced by *convolutional layers*, which are inspired by the *local receptive field* of the brain’s sensory system [30]. They apply a set of trained image filter windows on the image. One filter window is convolved with the input image, introducing the concepts of *weight sharing* in neural networks and resulting in a feature map that can be subject to a successive convolutional layer. Thus, a hierarchy of features is learned that represent different semantic abstractions of the input image. In contrast to hierarchical feature sets, CNNs are feature extractors and classificators combined, allowing end-to-end training from input sensor data to classification results. Especially, in cases like ours, in which the task of finding or extracting features is hard, CNNs can be used to find and extract features that fulfill this task. We apply this trait to find an intensity-based feature that separates surface plasmon resonance signals of differently sized bound nano-particles. As a loss function to minimize we use the cross-entropy

(1) $$L\left(f\left(x\right),y\right)=-\sum_{i=1}^{n}y_{i}\cdot \ln\left(f{\left(x\right)}_{i}\right),$$

so that the network learns to calculate a discrete distribution over n=27 particle size classes. For ground truth vector y, we use a normalized, discrete Gaussian distribution with the true particle size of the input particle as mean. The network is trained using the back-propagation algorithm and stochastic gradient descent [31], using synthetic training data for 23 classes (particle sizes 80 nm, 90 nm, ..., 290 nm, 300 nm) generated and interpolated from small samples of real sensor data, based on theoretic assumptions about the sensor signal [1,18]. When applying the trained network for estimation of particle size in the outlined image processing pipeline, we calculate the expectancy value of the neural network’s output

(2) $$s=\sum_{i=1}^{n}\left(c_{i}\cdot f{\left(x\right)}_{i}\right)$$

with label vector $c={\left(60,70,...,310,320\right)}^{⊤}$ for each individual signal. We then count the particles of different sizes and create the output distribution.

## 3. Results and Discussion

### 3.1. PAMONO-Sensor Enables Detection of Microvesicles (MVs) Derived from SH SY5Y Cells

**Figure 3:**
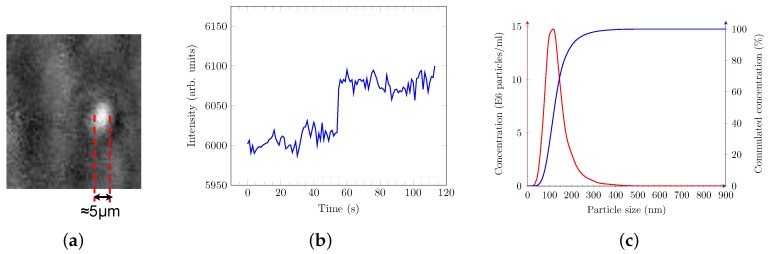
Typical processed image of a microvesicle binding onto the functionalized sensor surface is represented by a bright spot on a grey background (**a**). Bright pixels grouped in one spot stand for one binding event. Time-course changes of the light intensity in such a group of pixels are described by a vertical jump of intensity, in a moment of particle binding, at a new oscillation level and stabilization there (**b**). In parallel, we also measured the samples containing microvesicles derived from SH-SY5Y cells using LM10 device. The results of one of such measurements are presented on panel (**c**). The uniqueness of a significant particle size peak and similarity of the measured particle size with previously published results indicate that samples measured by our PAMONO-sensor consist predominantly of individual microvesicles. (**a**) sensor image data; (**b**) intensity step over time; and (**c**) LM10 measurement.

In previous studies [9], we used biotin-thiol, streptavidin and biotinylated anti-analyte antibody self-assembling layers for sensor surface functionalization. However, this approach has several disadvantages: necessity to use biotin-conjugated antibodies, single-use gold sensor and a lack of oriented immobilization of anti-target antibody onto sensor surface, which, in turn, can affect the efficiency of targeted vesicle capture. As a means of overcoming these issues, we tested here the use of PAMONO-sensor surface functionalization with cysteine-conjugated protein A/G. A similar approach utilizing cysteine-conjugated protein G was already successfully used in conventional SPR studies dealing with the formation of layers of biomolecules on the sensor surface [32]. However, this procedure has never been employed to image individual MVs or VLPs. After recording of images using *Streampix 3.0* software (NorPix, Montreal, Quebec, Canada), the processing of images was performed using the software developed by the authors [17,18,19]. A typical processed image of a bound MV is presented in Figure 3a. Binding events are also characterized by the changes of reflected light intensity over time, which appears as a step function Figure 3b: relatively flat intensity before the binding event, bounce of the intensity level during a binding event and further intensity oscillation around a new flat level. The samples containing MVs were also analyzed by Nano-particle Tracking Analysis (NTA) using the LM10 device (Malvern Instruments Ltd., Malvern, UK via Malvern Instruments GmbH, Herrenberg, Germany). These measurements revealed only one significant and practically normally distributed peak with a mean size value around 117±6 nm (Figure 3c). This is in good agreement with other studies showing a mean diameter of MVs from SH-SY5Y cells around 93–99 nm [33]. Moreover, the normal mono-distribution is indicative for a sample containing only individual (not aggregated) MVs. In turn, the latter fact states that the samples analyzed by the PAMONO-sensor consist predominantly of individual microvesicles.

### 3.2. PAMONO-Sensor Measurements Permit to Compare Relative Microvesicle Concentrations in Samples

**Figure 4:**
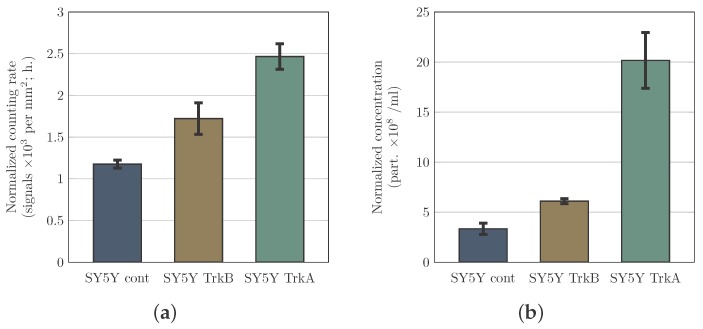
Comparison of samples containing microvesicles derived from non-transfected SH-SY5Y cells and cells transfected and expressing either TrkA or TrkB. Samples were measured using the PAMONO-sensor (**a**) and LM10 device (**b**). Microvesicle concentrations were detected by the LM10 device and demonstrated the following tendency—normalized microvesicle counts were increasing from non-transfected cells (SY5Y cont.) to cells expressing TrkB (SY5Y TrkB) and were the highest by cells expressing TrkA (SY5Y TrkA). These LM10 measurements were performed at least in triplicates and served as reference measurements. The same trend was received for counting rate measurements performed with the same samples using the PAMONO-sensor. Four measurements were performed using two non-crossing sensor regions in combination with two non-crossing time intervals. Error bars represent SEMs. These results demonstrate that the PAMONO-sensor allows for comparison of relative concentrations of microvesicles using only information about signal counting rates. (**a**) PAMONO-sensor; and (**b**) LM10 device.

It was an exciting task to check whether a very swift comparison of the relative microvesicle (MV) concentrations in two or more samples can be performed without a preceding calibration of the PAMONO-sensor. Recently, we demonstrated the linearity of calibration between concentration and counting rate for HIV-VLPs [9]. Therefore, we hypothesized here that samples containing MVs can be compared with each other even without establishing a calibration curve. This comparison can be based only on a difference between PAMONO-sensor counting rates of investigated types of MVs. A collation of Nano-particle Tracking Analysis (NTA) and PAMONO measurements supports this idea. We compared the results of PAMONO measurements in terms of counting rate with the results of NTA measurements, which provide concentrations of MVs. In both cases, the results are normalized to the number of cells producing MVs. These results are presented in Figure 4. Indeed, we demonstrate that measurements performed using the PAMONO-sensor (Figure 4a) can help quickly compare samples containing microvesicles. The results of the PAMONO- sensor are qualitatively in a good agreement with the results of NTA performed on LM10 device and serving as reference measurements (Figure 4b). Thus, a fast qualitative comparison of the MV containing samples can be performed using the PAMONO-sensor functionalized with an anti-CD81 antibody. It is also worth mentioning that the PAMONO-sensor provides MV-specific information since signals appear from the particles captured by anti-CD81 antibody. CD81 is known to be enriched on extracellular vesicles (microvesicles and exosomes) from different cells and a majority of extracellular vesicles (EVs) express this marker. Thus, it is often used as an indicative marker of EVs [12]. A certain quantitative disagreement between the results of the PAMONO-sensor and NTA measurements may have a biological nature. It is possible that overexpression of TrkA by SH-SY5Y cells significantly affects the level of surface cellular and, consequently, MV-associated CD81. This is an enthralling hypothesis, which certainly can inspire further studies. However, our current research work is focused on the features of the PAMONO-sensor and, thus, cannot be devoted to the deep investigation of the mentioned hypothesis.

### 3.3. Estimation the Specificity of Vesicle Detection by the PAMONO-Sensor

For these experiments, two HIV-VLP pseudotypes were produced: particles with and without ovalbumin (OVA) located on the surface. These VLPs are designed as a useful model to study antigen-based specificity of detected signals. The sensor surface was functionalized with an anti-OVA antibody giving an opportunity to estimate this specificity. Evaluation of antigen-based specificity has been previously addressed in our studies [9]. However, in the earlier work, another type of sensor surface functionalization was employed (biotin-thiol-streptavidin-biotinylated antibody) and the corresponding concentrations of VLPs were determined using an indirect method—Enzyme-linked Immunosorbent Assay (ELISA). Both issues motivated us to re-estimate the selectivity of signals detected by the PAMONO-sensor. In the present work, we performed PAMONO-sensor measurements employing cystein-conjugated protein A/G sensor surface functionalization and calculated concentrations of HIV-VLPs using LM10 device (Malvern Instruments Ltd., Malvern, UK via Malvern Instruments GmbH, Herrenberg, Germany). LM10 is based on the principles of nano-particle tracking analysis (NTA) and, thus, represents a direct optical method for the determination of particle concentrations. In order to compare two pseudotypes of Human Immunodeficiency Virus Virus-like Particles (HIV-VLPs), concentrations ascertained by LM10 were used to normalize the number of signals received during PAMONO-measurements. The results of our experiments are presented in Table 1.

Being confined by the availability of HIV-VLPs, we focused on the experiments with protein A/G sensor surface functionalization (four experiments performed) and made only one verification experiment with biotin-thiol-streptavidin functionalization. Our results show that a quantity of HIV-VLPs with OVA produces approximately ten times more (see Table 1 for details) signals then the same number of particles without OVA placed in the same volume. This means that, in a 1:1 mixture of VLPs with and without OVA, about 90% of the detected signals should correspond to the particles with OVA. Furthermore, both types of sensor surface functionalization provide similar sensitivity to particles, presenting ovalbumin on the surface, and, thus, are comparable in their power to capture target-particles. The difference between estimated specificity values demonstrated in the present study and, in our previously published work, [9] can be associated with an application of ELISA measurements for determination of particle concentrations in the previous work. ELISA is an indirect method, which does not permit calculation of concentration of particles without its recalculation from protein concentration. Such an approach can lead to a significant inaccuracy in calculations. NTA analysis performed here allows directly quantifying the number of particles in samples and, thus, can provide more accurate information on concentration of particles than ELISA. In turn, this information is important for normalization, which is performed in our present and previous works, and which affects the estimation of specificity of detected signals. It is noteworthy to mention that current estimation of specificity is carried out without any additional blocking of potential non-specific binding sites onto a functionalized sensor surface. Thus, an application of special blocking approaches gives an opportunity to improve current sensor specificity.

### 3.4. Evaluation of the Changes in the Counting Rate of HIV-VLPs after Repeated Use of the Same Gold Sensor

**Figure 5:**
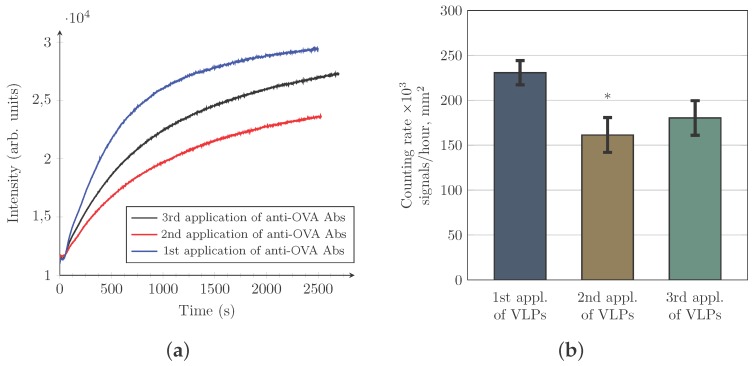
Changes of virus-like particle (VLP) counting rates after repeated use of the same gold sensor were investigated. Sensor surface is functionalized with cysteine-conjugated protein A/G and further covered with anti-ovalbumin antibody. After the first coverage with antibody and a measurement of VLP counting rate, an elution of antibody layer is performed. Then, after recovery with Phosphate-buffered saline (PBS) buffer, the second time coverage with anti-ovalbumin antibody and again a measurement of VLP counting rate are carried out. This cycle is repeated the third time. Three independent experiments were performed. The efficiency of antibody binding to the sensor surface is monitored and data are presented on the graph (**a**). The typical monitored antibody binding curves are displayed (for one experiment from three performed). Counting rates of HIV-VLPs are presented on the graph (**b**). Statistical analysis was performed using Student’s *t*-test. Significance was set at p<0.05 and marked with symbol “*” (significant difference between the first application of VLP and the second). (**a**) antibody binding efficiency; and (**b**) HIV-VLP counting rates.

Functionalization of the sensor with cysteine-conjugated protein A/G opened an attractive opportunity for the repeated use of the same gold sensor slide. Thus, the antibody layer (composed of the same or different antibodies) may be formed onto the protein A/G layer once again. However, it remained unclear whether a particle counting rate will stay unchanged after several re-use cycles of a gold sensor. To investigate this question, we used HIV-VLPs possessing ovalbumin (OVA) on their surface. We functionalized a gold sensor with cysteine-conjugated protein A/G and further with anti-OVA antibody. The results of our experiments are presented in Figure 5. Indeed, we are able to cover the gold sensor surface with anti-OVA antibodies up to three times using coverage/elution cycle. The efficiency of antibody binding to the gold sensor surface decreased around 22% during the second round of the antibody coverage/elution cycle (Figure 5a). During the third cycle, this efficiency remains practically unchanged. It is important to mention that, at all times, equal concentrations of anti-OVA antibody were used to cover sensor surface. We also revealed that HIV-VLP counting rate decreased about 30% during the second application of VLPs and did not alter significantly during the third application of these vesicles (Figure 5b). Aliquots containing equal concentrations of HIV-VLPs were used all times (concentrations were checked using NTA). The appeared reduction in HIV-VLP’s counting rate may be associated with a certain type of initial loss of antibody binding sites by protein A/G during the first contact with an elution buffer. Then, after washing with PBS, a recovery occurs and the cysteine-conjugated protein A/G layer becomes less sensitive to the next application of the elution buffer. Another explanation is associated with a possible removal of non-tightly bound cysteine-conjugated protein A/G molecules from the sensor surface. After the first elution and recovery cycle, only tightly bound molecules remain and can serve for an antibody binding.

### 3.5. Monitoring of Particle Size Distribution Using the PAMONO-Sensor

**Figure 6:**
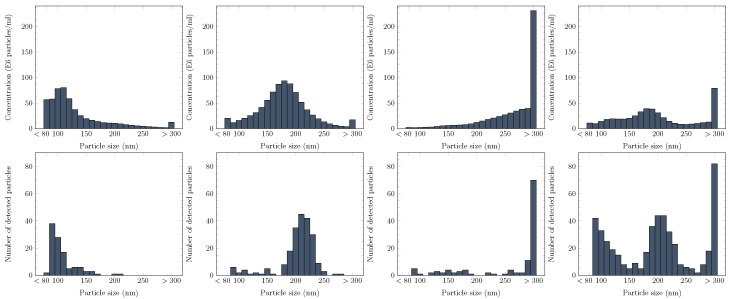
Experiment results for four different suspensions analysed by the PAMONO-sensor. The order of results for suspensions is the following (from left to right): solo 100 nm particles, solo 200 nm, solo 300 nm, the mixture of 100 nm and 200 nm and 300 nm particles. The top row shows reference distributions obtained with the LM10 device while the bottom row shows PAMONO-sensor results.

Nano-particle size distributions are derived with the image processing pipeline outlined in Section 2.4. We present result distributions for four different experiments in Figure 6. Three of the four measured liquids were compiled with one target particle size (100 nm, 200 nm, 300 nm) while the fourth one was a diluted mixture of these three. As reference distributions, we use particle concentration measurements performed by the LM10 device (Malvern Instruments Ltd., Malvern, UK via Malvern Instruments GmbH, Herrenberg, Germany). Since we trained our model for particle size classes from 80 nm to 300 nm, we modified the reference distributions accordingly: all concentration values for particle sizes s≤80 nm and s≥300 nm were added to the bin for 80 nm and 300 nm, respectively. This complements the fact that we trained our model to classify these out-of-range particles as particles of the border class, in order to avoid extrapolation with the trained model (which would not be reliable).

We outline three observations we made while comparing the distributions: First, our distributions contain more noise than the reference distributions. This is explained by the fact that we only see a small sub-sample of the whole set of particles present in the analysed suspension. As expected, we noticed a reduction of noise when increasing the number of analysed images. Second, we observe a particle size estimation error of 20–30 nm for particles larger than 100 nm. The origin of this error lies in our training data synthesis procedure. We lose image intensity in training data because of small translation invariances between different real data samples. However, this is a calibration problem and can be adjusted. Finally, our distributions show smaller standard deviations than the reference distributions, leading to more precise particle size predictions. For the 200 nm polystyrene particle suspension, the provider specified a particle size standard deviation of 9 nm. With our estimated distribution, we managed to close up to this value, compared to the reference distribution. In addition, it is important to note that a clear distinction between the three different particle classes is possible with the features we extracted. Based on the results, it is evident that we found extractable information in the SPR signals that enables particle size estimation of individual, bound nano-particles and, therefore, allows the automatic, real-time extraction of size distributions for a whole suspension sample.

We would still like to mention that our current results serve as a basis for the further adaptation of the PAMONO-sensor software for the size distribution measurements of biological nano-vesicles (microvesicles, for example). Certainly polystyrene particles, primarily due to the difference in the refractive indexes between them and biological microvesicles, cannot serve as etalon reference beads in biological measurements. Another type of biological vesicles of different sizes (for example, artificially prepared liposomes) may be helpful to perform an appropriate training of tested neural networks before their application in studies of microvesicle size distributions. However, it is also worth mentioning that the training procedure of the suggested model is not time-consuming and does not require a big number of images for training. The latter issues make realistic the employment of the described neural network approach into the analysis of MV size distribution.

## 4. Conclusions

We demonstrate for the first time the ability of the PAMONO-sensor to perform a real-time observation of the binding of label-free individual microvesicles to the sensor surface functionalized by protein A/G and anti-target antibody. Moreover, we show that measurements of counting rates, performed by dint of PAMONO-sensor, enable a swift comparison of relative microvesicle concentrations. Altogether, these findings indicate that, after appropriate calibration, a PAMONO-sensor can perform direct concentration measurements of microvesicles without an additional labeling step. The latter issue indicates the existence of a very attractive application field for this type of plasmonic sensor. It can be used in quantification of microvesicles and also in their sorting for post-PAMONO analysis (utilizing features of protein A/G sensor functionalization). Indeed, we confirmed the ability to re-use a gold sensor slide at least three times in the case of protein A/G based functionalization of the sensor surface. Thus, potentially, microvesicles or other biological sub-micrometer particles captured by anti-target antibodies, and further eluted from the sensor surface, can be processed to post-PAMONO analysis of their content and/or surface compounds. This issue is especially important for the clinical diagnostic field, where the content of extracellular vesicles and, particularly, microvesicles help to characterize the degree of disease progression. Moreover, we also demonstrated that nano-particle size information is evident in SPR signals and can be extracted by automatic, real-time processing of PAMONO-sensor images. Therefore, it is possible to estimate particle size distribution of suspension samples. Taken together, these proved that features of the PAMONO-sensor make it a great candidate for monitoring of concentration and characterization of microvesicles derived from eukaryotic cells.

In future work, these traits can be applied to develop a diagnostic platform based on the PAMONO-sensor, dealing with not only a simple quantification of microvesicles in liquid biopsy samples, but also with another crucial characteristic—microvesicle size distribution pattern. The latter issue provides additional information, since even being equal in number, but differing in sizes, microvesicles can carry drastically dissimilar cargo.

## Figures and Tables

**Table 1:** Determination of detection specificity using HIV-VLPs (human immunodeficiency virus virus-like particles).

	Protein A/G	Biotin-Thiol
**Ratio:** VLPs (virus-like particles) with OVA (ovalbumin) to VLPs without OVA (normalized counting rates were used)	12.6±3.9	21
Specificity (%)	88–94	95

## Abbreviations

The following abbreviations are used in this manuscript:

CNN	convolutional neural networks
ELISA	enzyme-linked immunosorbent assay
EV	extracellular vesicle
FBS	fetal bovine serum
FACS	fluorescence-activated cell sorting
HIV	human immunodeficiency virus
MV	microvesicle
NTA	nano-particle tracking analysis
OVA	ovalbumin
PAMONO	plasmon-assisted microscopy of nano-objects
PBS	phosphate-buffered saline
SPR	surface plasmon resonance
STR	short tandem repeats
VLP	virus-like particle
